# The Potential of Chronotherapy and Nanotherapy-Based Strategies for Glioblastoma Treatment

**DOI:** 10.3390/pharmaceutics18020235

**Published:** 2026-02-12

**Authors:** Ana Raquel Neves, Rafael Mineiro, Telma Quintela, Diana Costa

**Affiliations:** 1 RISE-Health, Department of Medical Sciences, Faculty of Health Sciences, University of Beira Interior, Av. Infante D. Henrique, 6200-506 Covilhã, Portugal; a34643@ubi.pt (A.R.N.); tquintela@fcsaude.ubi.pt (T.Q.); 2 BRIDGES—Biotechnology Research, Innovation and Design for Health Products, Polytechnic University of Guarda, 6300-559 Guarda, Portugal

**Keywords:** chronotherapy, circadian rhythm, clock genes, gene therapy, glioblastoma, nanotechnology

## Abstract

Glioblastoma is the most common and aggressive brain tumour in adults, and despite ongoing efforts, effective treatment remains limited. Standard therapies often face challenges because of the tumour’s specific biology, its aggressive nature, and the presence of certain physiological barriers in the brain that impede chemotherapeutics from reaching their target. Emerging research in circadian biology highlights the role of the internal circadian clock in tumour progression and treatment response. Evidence suggests that aligning therapy to patients’ chronotypes could potentially improve treatment outcomes. At the same time, advances in nanotechnology—including functionalized nanoparticles for drug and/or gene delivery—show promising results while reducing side effects. Additionally, evolving and prominent artificial intelligence tools may significantly contribute to progress in the design of next-generation personalised therapies. This review provides a unique and integrative perspective by examining the hurdles in treating GB and exploring innovative strategies, such as the integration of nanotechnology into chronotherapy protocols, to enhance therapeutic efficacy. The Chronobiology–Nanotechnology combination could not only improve GB patients’ survival rates but also lead to a more effective and less toxic personalised approach, distinguishing this work from previous reviews.

## 1. Introduction

Glioblastoma (GB) is the most prevalent and aggressive primary adult brain tumour, with poor prognosis and no effective therapies [1,2]. Standard treatments follow the Stupp protocol: surgery, radiotherapy, and temozolomide (TMZ) chemotherapy [1,3]. However, GB recurrence is very common due to its aggressive biology, rapid proliferation and migration, heterogeneity, and high infiltration into healthy brain tissue [1,2]. Furthermore, the blood–brain barrier (BBB)—responsible for controlling the nutrient and oxygen exchanges between blood and neural extracellular fluid to maintain normal brain function—and the tumour environment, make it difficult for chemotherapeutics to reach the brain and lead to therapy resistance [3,4]. Recent research highlights the role of circadian biology in tumour development and treatment [1,5].

Circadian rhythms are endogenous oscillations present in several biological processes across all organisms, and they have evolved over time to synchronise with the Earths’ rotation, helping organisms better adapt to their environment and survive [6,7]. Circadian rhythms are generated by the circadian timing system, which is synchronised primarily by light, through the central clock [1,5]. Additional environmental cues (zeitgebers), such as food intake, physical activity, geographical location, and temperature, further modulate these rhythms [1,5]. According to the time of the day, certain processes are activated or repressed, triggered by these environmental cues [1,8]. In mammals, after the retina receives light, the master clock present in the suprachiasmatic nucleus (SCN) signals the peripheral clocks to coordinate biological processes [1]. Circadian locomotor output cycles protein kaput (CLOCK), basic helix–loop–helix ARNT-like protein 1 (BMAL1), cryptochrome (CRY), and period (PER) are some of the core clock components that are involved in interlocked feedback loops as part of circadian mechanism regulation. Circadian rhythm disruptions caused by modern lifestyles (social jetlag, shift work, exposure to light at night, etc), along with genetic mutations or drugs, have been linked to some diseases’ development and progression [1,3,7]. In fact, several tumour-related genes are regulated by the circadian clock or exhibit circadian modulation of their expression, often linked to cell cycle control and DNA repair pathways [8]. Chronobiology investigates the circadian molecular mechanisms that regulate organisms in both normal physiological processes and pathological conditions [1,3]. Research in this field can influence therapeutic outcomes and side effects, for example, in the investigation of brain tumours [1,3]. Incorporating patients’ chronotypes (personal daily rhythm) into therapeutic plans would be a huge advance in the treatment efficacy of GB [1]. In addition, nanotechnology offers promising advances in glioblastoma treatment by enabling targeted drug delivery, improving therapeutic efficacy, and bypassing the BBB. This technology also aims to minimise side effects, enhance drug bioavailability and promote its sustained release, and increase drug accumulation at the tumour [9,10,11]. Cell-penetrating peptides (CPPs), polymeric nanoparticles, liposomes, inorganic nanoparticles, and exosomes have all been developed as vehicle systems to facilitate the transport of currently applied antineoplastic agents. Additionally, the use of gene therapy, involving the delivery of nucleic acids through delivery vehicles, has been shown to improve GB therapeutic effects [12,13,14,15,16].

This review mainly aimed to give insights into GB circadian modulation and an emerging therapy approach towards GB, chronotherapy, and its possible conjugation with nanotechnology. By discussing how these two strategies, alone or combined, enhance therapeutic efficacy, this work highlights the novel opportunities offered by integrating circadian biology and nanotechnology, providing a comprehensive and translational perspective that distinguishes it from previous reviews.

## 2. Glioblastoma

Glioblastoma is the most frequent adult brain cancer type (~50% of total cases) and is considered one of the most malignant of the tumours localised in the central nervous system (CNS), with a survival percentage rate of 4–7% [17,18]. The incidence in adults is 3.21 cases per 100,000 people in North America and Europe, but still considered a rare disease [19]. The mean age of diagnosis is approximately 64 years, and it is 1.6 times more prevalent in men than in women [9,19]. It was defined by the World Health Organization (WHO) in 2021 as a grade 4 astrocytoma, the highest grade for tumour malignancy [3,20]. Grade 4 tumours are characterised by their high heterogeneity, aggressiveness, resistance to treatment, poor prognosis, and low survival rate [20,21]. It originates from neural stem cells (NSCs), NSC-derived astrocytes, and oligodendrocyte precursor cells [3,15,18,20].

**Figure 1:**
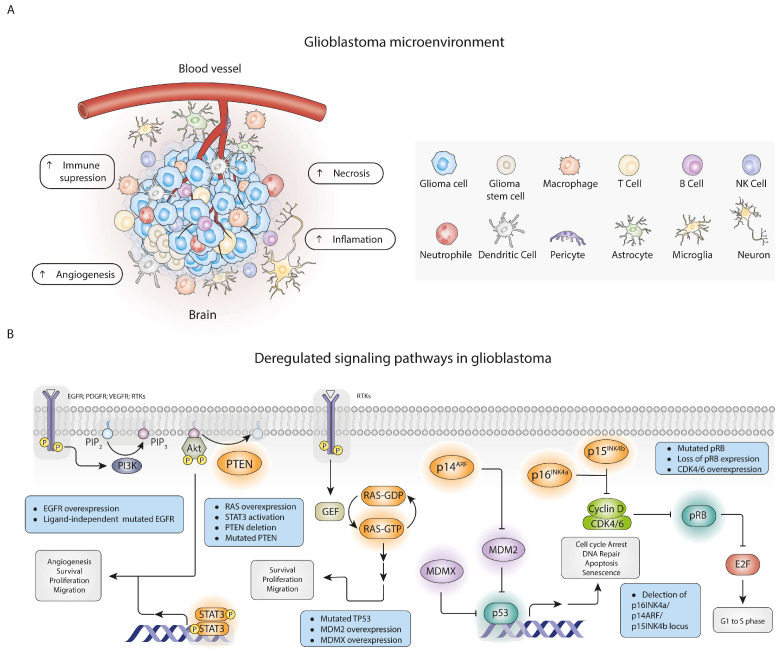
The complex biology of glioblastoma and deregulated signalling pathways: (**A**) The glioblastoma tumour microenvironment consists of a heterogeneous mix of cancerous glial cells, including astrocytes, and microglia, along with blood vessels and immune cells. The tumour is characterised by increased angiogenesis, immune suppression, inflammation, and necrosis, which is supported by the presence of abnormal blood vessels. (**B**) Key deregulated signalling pathways in glioblastoma. Alterations in cell-cycle control pathways, EGFR mutation, PTEN deletion, RAS overexpression, and STAT3 activation promote tumour development. This figure was partially composed using images retrieved from Servier Medical Art (Servier), licenced under the Creative Commons Attribution 4.0 International License (CC BY 4.0), Available online: https://creativecommons.org/licenses/by/4.0/ (accessed on 4 February 2026). T cell—T lymphocyte; B cell—B lymphocyte; NK—Natural killer cell; EGFR—Epidermal growth factor receptor; PDGFR—Platelet-derived growth factor receptor; VEGFR—Vascular endothelial growth factor receptor; RTK—Receptor tyrosine kinase; PIP—Phosphatidylinositol phosphate; PI3K—Phosphoinositide 3-kinase; Akt—Protein kinase B; PTEN—Phosphatase and tensin homologue; GEF—Guanine nucleotide exchange factor; RAS—Rat sarcoma small GTPase; GDP—Guanosine diphosphate; GTP—Guanosine triphosphate; ARF—ADP-ribosylation factor; INK—Cyclin-dependent kinase inhibitors; pRB—Retinoblastoma protein; CDK—Cyclin-dependent kinase; STAT3—Signal transducer and activator of transcription 3; MDM2—Mouse double minute 2 homologue; MDMX—Mouse double minute X homologue.

At the cellular/tissue level, histological analysis reveals extensive necrosis within the tumour, irregular cell and nucleus morphology, and abnormal blood vessel growth [17,20]. Furthermore, multiple cell populations, cellular states, and epigenetic modifications contribute to this high heterogeneity, angiogenesis, and phenotypic plasticity [9,22]. The GB tumour environment comprises the extracellular matrix (ECM), cytokines and chemokines, growth factors, astrocytes, neurons, pericytes, immune cells (microglia, macrophages, B cells, T cells, natural killer (NK) cells, neutrophils, dendritic cells), and glioma cells (GSCs), as illustrated in Figure 1A [9,22]. GB cells are also recognised for activating a series of tumour suppressor p53 signalling pathways, with the receptor tyrosine kinase (RTK)/Ras/PI3K signalling pathway, and the retinoblastoma pathway, contributing to a high proliferative rate and uncontrolled growth [9]. Methylation of O6-methylguanine-DNA methyltransferase (MGMT) promoter and mutations in isocitrate dehydrogenase 1 (IDH-1) and IDH-2 have been deeply associated with the aggressiveness of GB and the poor prognosis of patients [15,18]. MGMT-methylation status predicts response to DNA alkylating agents such as TMZ during chemotherapy [3]. Also, mutations in significant genes such as *TP53*, *NFK1*, *PTEN*, *EGFR*, *PDGFRA*, and *PIK3* contributed to tumour heterogeneity [20]. TP53 dysfunction is directly correlated with cancer initiation and progression [3]. Key cell-cycle and growth factor signalling pathways that are commonly deregulated in glioblastoma are summarised in Figure 1B.

Clinically, symptoms range from headaches to seizures, neurologic deficits, and personality changes [3,9,21]. Diagnosis focuses on biopsy results and imaging findings, normally by the magnetic resonance imaging (MRI) technique [3,9]. The last resource is usually applied at advanced stages of tumour development due to the difficulty of differentiating non-neoplastic lesions from GB [9]. This contributes to the lack of effective diagnosis approaches and delays in disease treatment [9].

## 3. Standard Care Treatments

Current therapeutic approaches have improved patients’ mean survival (14–15 months); however, prognosis and life quality are still very poor [18]. As a first line, the patient is subjected to the Stupp protocol and total tumour resection (when feasible), accompanied by radiotherapy and chemotherapy [3,18,21]. Radiotherapy may include several approaches and usually begins a few days post-surgery [10,21]. The safety of resection and radiotherapy efficacy depends on tumour imaging and diagnosis [3]. Sometimes, the location of a brain tumour makes complete surgical resection difficult, and because glioblastoma cells grow diffusely and tend to infiltrate normal brain tissue, cycles of chemotherapy are usually recommended after radiotherapy [10,11]. Unfortunately, estimates suggest that within two years, 90% of patients experience a recurrence of the tumour [9,18,23].

Chemotherapy mainly targets proliferating cells, but it is ineffective against core tumour cells [18]. Furthermore, tight junctions and adherence junctions between brain endothelial cells (ECs) from the BBB limit drug penetration and accumulation, contributing to chemoresistance development [9,11,17]. TMZ and bevacizumab are the two chemotherapeutic agents that make up part of Stupp’s protocol [20]. TMZ, approved in 2005, is a DNA-alkylating agent that methylates the O6 group from DNA guanine, leading to the formation of O6-methylguanine adducts [2,23]. Adduct formation then triggers futile cycles of mismatch repair, which repeatedly attempt to correct the DNA lesion [2,23,24]. Tumours with methylated MGMT promoter have increased sensitivity to TMZ, which leads to prolonged patient survival [23]. Myelosuppression, neutropenia, leukopenia, and cerebral oedema have been associated with TMZ intake [2,17]. Some concomitant and/or adjuvant therapeutic agents have been developed, and some have been approved to improve TMZ efficacy and prolong the survival of patients [2]. Bevacizumab was approved by the Food and Drug Administration (FDA) in 2009 and is a humanised monoclonal antibody that binds and neutralises vascular endothelial growth factor (VEGF) to block angiogenesis [2,9,21,25]. This antiangiogenic drug is used to treat radiation necrosis and symptomatic oedema and has been proven to enhance progression-free survival (PFS); however, it fails due to its short half-life and tumour immune invasion [2,9,17]. Hypertension, cerebral bleeding, and thromboembolic events are some side effects associated with this strategy [2]. In recurrent patients with rapid disease progression, bevacizumab can be administered in combination with Lomustine (CCNU) [26]. CCNU and carmustine (BCNU) are non-specific alkylating agents that link to DNA and RNA, leading to cell death [2]. Hematologic toxicity and bone marrow suppression are significant adverse effects linked to CCNU use, along with ocular and pulmonary toxicities associated with BCNU administration [2]. Other agents used include nitrosourea, gefitinib, aflibercept, irinotecan, procarbazine, and vincristine (PCV) [10,21]. However, these drugs exhibit low bioavailability, poor pharmacokinetics, and cause brain tissue damage [11].

## 4. Chronotherapy

Chronotherapy has emerged as a promising approach in cancer treatment, since it may optimise treatment effectiveness while reducing side effects [3,7]. It consists of the synchronisation of clinical interventions, such as medication, nutrition, light exposure, and other therapies, with the natural rhythms of patients’ circadian biology [5].

The brain’s central role in regulating systemic circadian rhythms has led to a recent focus on the molecular circadian clock within brain tumours [1]. Understanding how these tumour-specific clocks interact with central circadian signals has revealed opportunities to directly modulate the clock and treat disorders associated with circadian rhythm dysfunction, both by identifying new targets within clock components and by modulating previously characterised molecular pathways [7]. By linking central and tumour-specific clocks, this approach provides a clearer understanding of how systemic circadian signals influence tumour biology and therapeutic responses.

### 4.1. How the Circadian Clock Works

**Figure 2:**
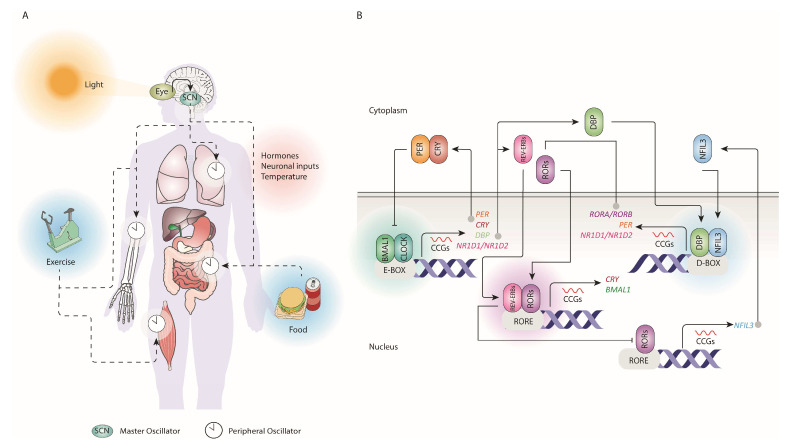
The internal clock controls daily biological rhythms: (**A**) Light and other external signals reset the central clock in the SCN of the hypothalamus, which synchronises peripheral clocks across the body. (**B**) Circadian clocks operate through key molecular components and regulatory pathways. This figure was partially composed using images retrieved from Servier Medical Art (Servier), licenced under the Creative Commons Attribution 4.0 International License (CC BY 4.0), Available online: https://creativecommons.org/licenses/by/4.0/ (accessed on 2 February 2026). CCG—Clock-controlled gene; DBP—D-box-binding protein; NFIL3—Nuclear factor interleukin 3 regulated.

Circadian rhythm refers to the ~24 h daily fluctuations in physiological mechanisms synchronised with the Earth’s diurnal cycle [7,27]. This rhythm is present in all living organisms, and it represents an evolutionary adaptation to environmental changes [7]. The circadian clock is the endogenous biological clock present in all organs and individual cells (peripheral clocks) that commands and generates the circadian rhythm [27]. External and environmental stimuli named zeitgebers, like light, temperature, exercise, eating patterns, and socialisation, influence circadian rhythm synchronisation, as represented in Figure 2A [7,8,27].

All internal circadian clocks in the individual cells are controlled by a major clock located in the SCN of the hypothalamus [27]. As the light is the principal zeitgeber, the SCN receives light from the retinal ganglion cells and thereby synchronises all peripheral clocks, regulating biological functions according to the diurnal period (Figure 2A) [1,7,27].

Circadian rhythms are driven by core clock proteins operating within translational–transcriptional feedback loops (TTFL) [3,23,28]. Figure 2B presents an overview of the clock molecular network. CLOCK and BMAL1 are considered the master clock regulators [1]. Within the positive loop, the BMAL1/CLOCK complex modulates the transcription of *PER* (*PER1*, *2*, *3*), Nuclear Receptor Subfamily 1 group D (*REV-ERB*) (*REV-ERBα*,*β*), *CRY* (*CRY1*, *2*), and *ROR* (Retinoic Acid Receptor (*RAR*)-related Orphan Receptor) (*RORα*,*β*,*γ*) by binding to the E-box enhancer element present in their clock-controlled gene (CCG)-promoter regions [1,3,27,28]. In the negative loop, PER and CRY proteins accumulate in the cytoplasm and heterodimerize, enter the nucleus, and repress *BMAL1* and *CLOCK* transcription [1]. ROR, on the other hand, binds to the retinoic acid-related orphan receptor response element (RORE) on the promoter of *BMAL1* to activate *BMAL1* transcription, and REV-ERB protein competes to bind to the same promoter to inhibit *BMAL1* transcription [1,3,28].

In the event of a deviation from the typical daily routine, the circadian rhythm is known to be adversely affected. A number of diseases have been linked to these disruptions, including cancer [3,7,8]. Shift work, jet lag, exposure to artificial light after sundown, and drug intake are risk factors associated with circadian rhythm disruption [1,7]. Recent studies have revealed an emerging understanding of how circadian biology contributes to tumorigenesis, disease progression, and resistance to treatments [5,29,30,31]. Importantly, while these environmental and behavioural factors can disturb patients’ circadian rhythms, tumours often exhibit their own intrinsic biological clocks, which may be partially dissociated from systemic rhythms [31]. Unravelling the crosstalk between host and tumour clocks paves the way for incorporating circadian medicine into several diseases, ranging from cardiovascular diseases to cancer [5,29,30].

### 4.2. Circadian Clock Disruption: Implications on GB

#### 4.2.1. Influence on Tumour Microenvironment (TME)

The dynamic nature and heterogeneity of high-grade glioblastomas are partly due to their capacity to modify and adapt cell homeostasis and the tumour microenvironment (TME) in response to surrounding factors [23,32]. As glioblastoma cancer cells create an immunosuppressive environment around the tumour, immunotherapies often show limited efficacy [23]. Circadian clock disruption has been demonstrated to have a significant impact on tumour growth, proliferation, angiogenesis, immortality, the ability to evade apoptosis pathways and growth suppressors, and the TME, through the activation of inflammatory, immune suppression, angiogenesis, and oncogenic signalling pathways [23,32,33,34,35]. Targeting circadian clock elements involved in such mechanisms may assist in the reversal of the pathogenesis of TME and glioblastoma [23]. Below is a more detailed description of several studies investigating the relationship between circadian clock disruption and the mechanisms associated with glioblastoma tumorigenesis.

#### 4.2.2. Influence on Angiogenesis

GB angiogenesis is a well-known hallmark of this kind of cancer [28]. GB has the ability to alter its TME via angiogenesis by activating certain angiogenic factors, such as the VEGF, leading to the formation of new blood vessels around the tumour to suppress energetic demands and maintain continuous growth and proliferation [23]. Pang et al. demonstrated that this process can be dependent on circadian regulation and specific circadian gene expression, and it is associated with higher BMAL1 expression in glioma cells [36]. Indeed, *CLOCK* or *BMAL1* knockout resulted in tumour progression inhibition and angiogenesis [36]. Activation of REV-ERBα, by the SR009 administration, caused a decrease in this via and blood vessels formation in vivo [36]. In another study, in high-grade glioma patients, pro-angiogenic factors were positively correlated with BMAL1 expression [37].

#### 4.2.3. Influence on Cell Proliferation, Invasion, and Migration

The ability of high-grade glioma cells to proliferate, migrate, and invade distant tissues has already been associated with the circadian clock [23,28]. CLOCK was shown to stimulate glioblastoma cells’ migration by increasing the transcription of the nuclear factor-κB (NF-κB) [38]. Additionally, Yu et al. tested the influence of REV-ERBβ suppression on glioblastoma cells’ migration and discovered a positive impact on cell metastasis progression [39]. RORα, PER2, and BMAL1 were also shown to have a positive impact on cell proliferation [40,41,42,43,44].

#### 4.2.4. Influence on Inflammation

Glioblastoma is recognised for its strong immunosuppressive properties [28]. The circadian clock has a key role in cytokine expression and immune system regulation within the TME [23]. The levels of proinflammatory cytokine interleukin-1ß (IL-1ß), IL-8, IL-6, and lactate dehydrogenase A (LDHA) were seen to decrease after CLOCK and BMAL1 expression suppression in glioma cells, which revealed the existence of a lactate-IL-1β-Clock (LIC) loop involved in tumour progression and inflammation [45]. BMAL1 overexpression and RORα low levels were, in another study, linked to the attraction of suppressor cells and cells in the immune system with infiltration abilities [46]. Additionally, other studies highlighted the impact and importance of the circadian clock in the molecular mechanisms involved in immunosuppressive TME promotion [43,44,47,48].

#### 4.2.5. Influence on Immune System

A suppressed immune system associated with gliomas has recently been linked to BMAL1, CLOCK, and CRY1/2 circadian protein dysregulation [23,28]. A study from Nelson et al. showed that microglia, myeloid-derived suppressor cells (MDSCs), and regulatory T cells in GB are influenced by the circadian clock (especially via CLOCK/BMAL1) [28]. Chen et al. demonstrated that CLOCK in GSCs upregulates a chemokine, OLFML3, attracting microglia into the GB tumour microenvironment [47]. Building on this, Xuan et al. demonstrated that the BMAL1/CLOCK complex induces OLFML3, which, via hypoxia-inducible factor 1-alpha (HIF-1α) signalling, increases legumain (LGMN) and CD162 in GSCs [48]. This pathway promotes the recruitment of microglia while reducing CD8^+^ T cell infiltration and activity [48]. CRY1/2 also appears to have a role in immunosuppression in GB TME by negatively regulating the activity of HIF-1α [49].

So, CLOCK, BMAL1, and CRY1/2 modulation can significantly shape the immune landscape, increase the gliomas’ responses to immunotherapies, and prolong survival [23]. For example, REV-ERBα agonists that can inhibit BMAL1 have been proven to reduce immunosuppressive microglia in GB models [23]. Also, blocking the CLOCK-OLFML3-HIF-1α–LGMN–CD162 pathway, combined with the administration of inhibitors timed to coincide with peak T cell function, microglial activity, and cytokine signalling within the circadian cycle, may lead to optimised immunotherapy strategies. The use of immune checkpoint inhibitors (ICI) and cellular therapies as chimeric antigen receptor (CAR)-T cell therapy has also been revolutionising the cancer immunotherapy field in recent years [50,51].

#### 4.2.6. Influence on Apoptosis and Survival

p53 expression has been found to be deregulated in the majority of GB patients, thus enabling the cancer cells to effectively evade apoptosis mechanisms [28]. BMAL1/CLOCK has been shown to have a positive effect on this outcome, in contrast to CRY2, PER1, and RORα, which negatively influence cancer cells’ survival [28]. Nonetheless, research has demonstrated that glioblastoma cells can selectively activate survival pathways, enabling them to resist apoptosis, adapt to microenvironmental stress, and evade therapeutic interventions [28].

#### 4.2.7. Influence on Immortality and Stem-like Properties

The ability of cancer cells to exhibit stem cell characteristics is associated with therapy resistance and tumour recurrence [28]. PER2 and RORα were found to reduce stemness [42,43,44]. Chen et al. conducted a study demonstrating that BMAL1/CLOCK also has a role in the stemness process in glioma stem cells by inducing glucose- and fatty acid-metabolism-involved protein expression [47]. Indeed, conjugate suppression and CLOCK-regulated LGMN signalling pathway inhibition led to control of microglia biology and immunosuppression in GB mouse models [47].

#### 4.2.8. Circadian Clock Disruption: Paediatric High-Grade Gliomas

Also, in paediatric GB cases, the standard protocols designed for adult cancers fail to fight the tumour, and the outcomes remain very poor. Recently, dysregulation of circadian genes has also been associated with children’s brain tumours, such as GB and low-grade gliomas. Studies have shown that paediatric GB differs from adult gliomas in both tumour location and molecular subtype [52]. A study on hospitalised paediatric patients with medulloblastoma demonstrated signs of circadian rhythm dysregulation associated with greater fatigue [53]. In another study, the observation that *BMAL1*, *CLOCK*, and *REV-ERBα* expression are disrupted in paediatric high-grade glioma lines, with TMZ sensitivity varying according to the circadian cycle and BMAL1 levels, underlines the potential relevance of circadian biology in paediatric treatment response [54]. The same authors reported that high *BMAL1* expression in paediatric glioma patient data sets correlated with worse overall survival (OS) and upregulation of stemness-associated markers [55].

As they are different in nature, paediatric tumours would benefit from deeper research into chronotherapy in order to increase survival outcomes and quality of life.

### 4.3. Chrono-Based Approaches

Understanding how circadian rhythms influence disease has given rise to two main therapeutic strategies within circadian medicine. The first strategy aims to correct disruptions in the internal clock by directly restoring or modulating misaligned circadian rhythms [56]. In this context, approaches include adjustments in behavioural patterns, the administration of chronobiotics, and targeting core clock genes and signalling pathways [56]. Importantly, these interventions should consider the crosstalk between individual rhythms to achieve systemic and tissue-specific circadian alignment. The second approach, chronotherapy, aims to synchronise conventional drug administration with circadian oscillations [1,23]. Successful application of this strategy depends on both the drug’s pharmacokinetics and pharmacodynamics, as well as the circadian variation in the disease pathophysiology [56]. The goal is to optimise therapeutic outcomes by administering drugs at times when target pathways demonstrate peak responsiveness, disease activity reaches its highest levels, or drug tolerance is maximised [1,23,56]. In the end, it is important to determine whether a patient would respond favourably to a time-of-day-specific treatment plan beyond just selecting the drug and dosage to use [1].

In this section, an overview of current and emerging chrono-based approaches highlights both the limitations of existing protocols and the potential benefits of integrating circadian principles into GB treatment design.

#### 4.3.1. Clock-Related Components as Therapeutic Targets

Circadian clock components may act as innovative biomarkers in GB, with their expression patterns and/or circadian clustering providing valuable information on patient prognosis and treatment response [1]. A number of studies have demonstrated that the alteration of clock proteins can exert a substantial influence on tumorigenesis, progression, and risk of GB [28,56]. Therefore, circadian clock components have been explored recently in various studies as targets for GB treatment, since there is now a better understanding of the circadian clock’s role in cancer development, progression, and treatment.

##### BMAL1/CLOCK and PER2/3

A significant increase in *BMAL1* mRNA expression was observed in high-grade glioblastoma tissues [1,37]. In addition, elevated levels of *BMAL1* mRNA expression in grade III glioblastoma have been demonstrated to be associated with diminished survival periods, oedema severity, and an increased microvascular density [37]. On the other hand, studies suggested that the supplementation of BMAL1 via adenovirus reduced the growth, migration, and spread of U87 cells [57]. These findings indicate that BMAL1 status may serve as a valuable predictor of patient prognosis and responsiveness to anti-angiogenesis therapies. A different study employed a bioinformatic method using data on drug sensitivity and gene expression profiles, revealing that increased BMAL1 expression is strongly associated with sensitivity to TMZ [58]. The CLOCK expression has been observed to be elevated in 5% of cases, with the majority occurring in low-grade GB tumours [1,47]. Other studies suggest that the expression of BMAL1/CLOCK exerts a favourable influence on the proliferation of glioblastoma cells [45,59].

PER2 has been identified as a critical regulator of the stemness of GSCs, with evidence suggesting its role in downregulating the Wnt-β-catenin signalling pathway, proving PER2 to be a promising therapeutic target in the treatment of GB [42]. PER3 was expressed at low levels in GB tissue and was correlated with worse OS and prognosis, tumour infiltration, and immunotherapy response [60]. Experimental evidence further suggested that environmental factors, such as DEHP exposure, influenced PER3 expression and circadian dysregulation and promoted glioma development in a zebrafish model [60].

##### CRY1

Isoform-specific stabilisers of CRY regulators have been recently developed. A thienopyrimidine derivative, KL201, that selectively controls and stabilises CRY1, is a promising modulator for future chronotherapy strategies against clock-related diseases, since it has been shown to lengthen the period of cells’ and tissues’ circadian rhythms [61]. The development of other photoresponsive CRY1-selective molecules derived from benzophenone enabled reversible control of CRY1 function [62]. Regulation of CRY1 activity by photopharmacology could serve as a foundation for developing targeted chronotherapies. Despite the limited number of studies, the regulation of CRY family activity can positively contribute to therapeutic benefits in GB patients.

##### ROR-α

In a human glioblastoma cell line, LN229, ROR-α binding to the iron-chelating agent di-2-pyridylketone 4,4-dimethyl-3-thiosemicarbazone (Dp44mT), a potential drug for GB treatment, enhanced anti-oncogene N-myc downstream-regulated gene (NDRG)2 expression and led to inflammation suppression [44]. By indirectly regulating the miR-1290/RORA network, Sevoflurane (Sev) has been shown to block glioblastoma progression [40]. ROR-α overexpression is associated with proliferation and tumorigenesis blockage, achieved by means of the inhibition of the TNF-α-mediated NF-κB signalling pathway [43]. Furthermore, microRNA-18a was found to possibly be associated with ROR-α expression, thus making it a promising target to reverse the progression of GB [43].

**Figure 3:**
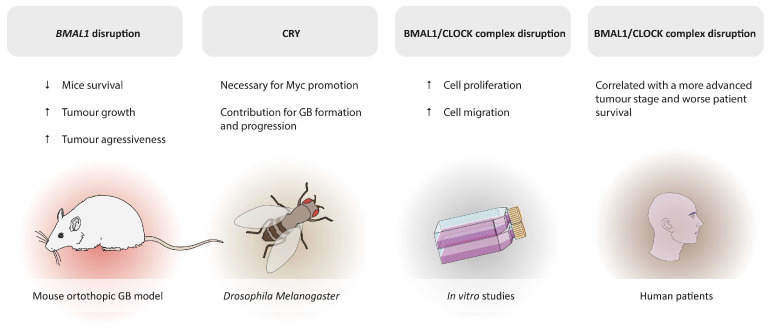
Impact of circadian clock disruption on glioblastoma progression across experimental models. This figure was partially composed using images retrieved from Servier Medical Art (Servier), licenced under the Creative Commons Attribution 4.0 International License (CC BY 4.0), Available online: https://creativecommons.org/licenses/by/4.0/ (accessed on 21 December 2025).

Figure 3 summarises additional studies supporting the association between circadian clock gene dysregulation and glioblastoma, emphasising the potential of clock-related components as therapeutic targets.

##### TIMELESS, DEC1, and NR1D1

Beyond the clock-related genes already discussed, several additional studies have explored how other circadian regulators, namely *TIMELESS*, *DEC1*, and *NR1D1*, influence GB development and progression. A study discovered that high TIMELESS expression defined a high-risk glioma subset associated with poorer OS [63]. *TIMELESS* knockdown reduced proliferation, altered expression of other clock genes, and induced G0/G1 cell cycle arrest, suggesting a role in promoting tumour growth [63]. Additionally, high TIMELESS expression correlated with the enrichment of immune and stromal pathways, linking circadian dysregulation to the tumour microenvironment [63]. In another study, authors analysed TIMELESS expression in glioma, and the results supported the pro-tumorigenic role of TIMELESS, acting through both cell-cycle regulation and the modulation of the tumour microenvironment [64]. DEC1 has also been associated with poorer prognosis in glioma patients [65]. Its overexpression in MGMT-positive glioma cell lines conferred TMZ resistance, whereas *DEC1* knockdown induced TMZ-apoptosis [65]. NR1D1 expression was found to be reduced in TMZ-resistant GB cell lines, yet higher NR1D1 levels in patient tumours have been associated with poorer prognosis [66]. These findings suggest that NR1D1 may play a significant role in modulating glioblastoma sensitivity to TMZ [66].

Targeting clock components, either directly, with small-molecule modulators, or indirectly, through chronotherapy approaches that synchronise treatment timing with circadian rhythms, may enhance treatment efficacy, reduce toxicity, and improve patient outcomes. Collectively, these findings indicate that integrating circadian biology into therapeutic strategies offers a promising avenue for glioblastoma.

#### 4.3.2. Chrono-Based Approaches in GB Therapy

Numerous clinical studies have found that administering treatments at certain times of day enhances positive physiological responses, boosts treatment effectiveness, and minimises toxicity [1,3]. Some other studies on rodents have indicated that the toxic dose of a therapeutic agent can be subject to 24 h variations in its absorption, distribution, metabolism, and excretion processes [3]. In fact, BBB permeability and xenobiotic efflux seem to be under the influence of the circadian clock, suggesting that CNS-targeted therapies may also be subject to time-of-day variations [67,68,69,70]. A study conducted by the Pulido research group showed that oscillations in P-glycoprotein (P-gp) efflux transporter function in brain endothelial cells were influenced by BMAL1 expression [67]. When BMAL1 was knocked out in these cells, the day/night rhythm in P-gp efflux became impaired [67]. Also, using quantitative proteomics, another study identified several proteins with diurnal oscillations involved in translation, endothelial signalling, and other BBB functions [68]. Zhang et al. did not observe strong oscillations in transcript levels of efflux transporters in endothelial cells, but found that BMAL1 regulates intracellular magnesium (Mg^2+^) levels via the magnesium channel TRPM7 [69].

As many genes and physiological pathways have proved to be under circadian rhythm influence, it is expected that drug absorption, metabolism, and efficacy may also fluctuate over the day. Key processes involved in pharmacokinetics can vary depending on the time of administration, meaning that the same medication taken at different times may produce different therapeutic effects, supporting the growing field of chronotherapy.

##### TMZ

TMZ has been the primary focus in the study of drug response over time. The combination of TMZ with a specific inhibitor of glycogen synthase kinase-3 (GSK-3) and casein kinase 1, CHIR99021, has shown increased efficacy in GB cell cultures when administered 18 h post-synchronisation, suggesting a time-of-day-dependent sensitivity [71]. A study involving 166 GB patients showed that the intake of TMZ in the morning produced longer OS compared to the evening, depending on the MGMT-methylation status of patients [72]. The authors performed a follow-up study with a phase II randomised clinical trial, which demonstrated that chronotherapy with TMZ is feasible [73]. A recent 2025 study compared the outcomes and toxicity in patients receiving TMZ in the morning, afternoon or evening [74]. While no association was observed between the timing of administration and OS, patients who received TMZ in the morning experienced higher rates of bone marrow toxicity [74]. Knowing that in humans, BMAL1 reaches its peak expression in the morning, it seems that maybe BMAL1 may play a key role in TMZ’s mechanism of action [3].

A study by Neves et al. explored a chrono-nanomedicine strategy that aligns drug/gene co-delivery with the circadian rhythms of GB cells to enhance therapeutic outcomes. The authors developed a WRAP5 peptide nanocomplex, functionalized with a transferrin receptor ligand, to co-deliver TMZ and a *TP53*-encoding plasmid to U87 glioma cells [75]. Circadian variations in clock genes (*Bmal1*, *Per2*) and transferrin receptor expression influenced nanocomplex uptake and gene transfection. Peak delivery times (T8, T16, corresponding to 8 and 16 h after cell synchronisation, respectively) significantly enhanced p53 expression. These findings highlight the potential of time-tuned administration to optimise TMZ/p53 efficacy and overcome GB resistance.

Given the limited number of preclinical and clinical studies exploring the influence of circadian rhythms on the therapeutic effects of TMZ, patient survival, and adverse effects, current findings remain inconclusive [28]. This also highlights the complexity of chronotherapy in glioblastoma. Discrepancies in findings may arise from several factors, including patient heterogeneity, differences in experimental or clinical study models, and the timing of TMZ administration. Future research should aim to elucidate the molecular mechanisms linking the GB circadian clock to drug response, standardise clinical trial designs, and investigate the mechanistic basis of time-dependent TMZ efficacy.

##### 1A-116 and Bortezomib

1A-116 is a therapeutic agent that has been investigated with regard to its proapoptotic and anti-invasive properties [3,76]. In a recent study on GB mouse xenografts, the application of small doses of 1A-116 around the expression peak of metastasis-inducing protein-1 (TIAM1) had similar effects when compared to higher doses administered at other time points [77]. The survival time of mice bearing the xenografts was also found to improve with administration at the end of the light cycle compared with the beginning of the light cycle, suggesting an influence and modulation of the circadian clock on 1A-116’s treatment efficacy [23,77]. The study also revealed that high expression of PER1 and lower expression of BMAL1 led to the best outcomes after 1A-116 administration [23,77]. Knockdown of *BMAL1* supported the idea of the circadian-regulation dependence of 1A-116 efficiency [23,77].

Bortezomib is an inhibitor of proteasomes, usually applied for multiple myeloma and lymphoma treatment [3]. Proteosomes are responsible for degrading PER and CRY when they accumulate [78]. A decline in PER and CRY protein levels reactivates BMAL1:CLOCK, triggering the onset of a new circadian cycle [79]. So, bortezomib could influence the oscillatory dynamics of circadian rhythms by repressing BMAL1 and CLOCK activity, which is strongly associated with glioma cell proliferation [80]. In mice bearing GB A530 glioblastoma cell xenografts, results suggest that administering bortezomib at night leads to higher treatment efficacy, even at low doses [81]. Furthermore, the co-administration of bortezomib with SR9009, a REV-ERB agonist, demonstrated enhanced cytotoxicity in T98G cells when applied during a circadian window centred 18 h after synchronisation, even at low concentrations [82]. This suggests a synergistic chrono-pharmacological effect [82].

Other chronotherapy strategies have been investigated in glioblastoma in order to optimise therapeutic effects while minimising toxicity. Table 1 provides additional chronotherapy strategies explored in the context of GB.

A comparison of the chronotherapy strategies presented in Table 1 highlights several key insights. TMZ chemotherapy showed pronounced circadian dependence, with maximal efficacy aligned with MGMT and BMAL1 peaks and morning administration improving tumour outcomes. Metformin combined with radiotherapy enhanced treatment sensitivity via PER2 and SIRT2/G6PD modulation, while dexamethasone exhibited daily rhythmicity, influencing glioblastoma progression. Immunotherapies, including anti-PD-1 with or without TMZ, generated oscillatory immune responses. Melatonin restored circadian rhythms under constant light and reduced tumour growth and vascularization. Finally, natural compounds such as *Lycium barbarum* extract inhibited proliferation and lipogenesis through PER2 upregulation. Collectively, these findings emphasise the critical role of circadian timing in therapeutic efficacy and support integrating chronotherapy into glioblastoma treatment strategies.

#### 4.3.3. Chronoradiotherapy

Evidence from multiple studies suggests that circadian physiology can affect radiotherapy outcomes and patients quality of life. In a range of cancers, circadian clock genes are intricately associated with immune-related processes [91]. Scheduling radiotherapy by time of day (morning or in evening), has been found to significantly influence treatment response [3]. Sapienza et al. initiated the study by administering radiotherapy to GB patients during morning or afternoon sessions [92]. However, authors did not find significant differences in toxicity and in PFS and OS, meaning that the time-based radiotherapy may not be significant when treating high-grade glioblastomas or that the designed time of day was not ideal for all of the groups under consideration [92]. Future studies should be conducted to thoroughly investigate this subject.

**Figure 4:**
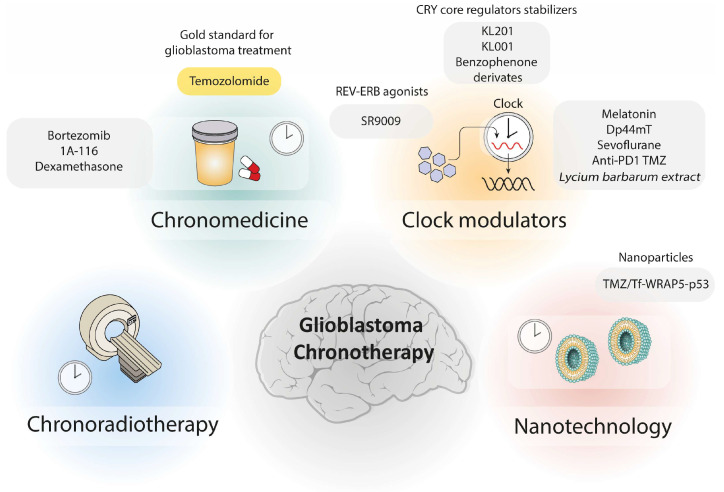
Illustration of glioblastoma treatment strategies using chronotherapy, which can help reset the biological clock, improving therapy effectiveness and outcomes. This figure was partially composed using images retrieved from Servier Medical Art (Servier), licenced under the Creative Commons Attribution 4.0 International License (CC BY 4.0), Available online: https://creativecommons.org/licenses/by/4.0/ (accessed on 2 February 2026).

Figure 4 provides an overview of chronotherapy-based therapeutic strategies currently explored for GB.

In summary, chronotherapy represents a promising approach for glioblastoma treatment by aligning therapeutic interventions with patients’ circadian rhythms. Tumour-intrinsic clocks, in addition to systemic circadian signals, influence glioblastoma progression, immune responses, angiogenesis, proliferation, and therapy resistance. Targeting circadian components such as BMAL1, CLOCK, PERs, CRYs, and RORα offers potential for improving drug efficacy, immunotherapy response, and patient outcomes. Chrono-based strategies, including time-tuned administration of TMZ, 1A-116, bortezomib, and nanomedicine, demonstrate that treatment timing can significantly affect therapeutic effectiveness and toxicity. However, these strategies differ substantially in their ability to achieve precise temporal control and efficient delivery to the tumour site.

Although these strategies offer considerable therapeutic potential, their implementation faces challenges. Direct clock-related component modulation repeatedly struggles with the delivery of chronobiotics to target tissues, as they must traverse physiological barriers [56]. Chronotherapy, especially when relying on systemic drug delivery, is restrained by the lack of precise temporal control over drug release and in vivo accumulation [56]. In this context, nano-based strategies emerge as particularly promising, as they can integrate temporal regulation with targeted delivery, thereby addressing key limitations of other chrono-based approaches. Hence, this review emphasises nano-based strategies as a promising avenue to overcome these limitations, further enhancing the translational potential of circadian-based glioblastoma therapy.

## 5. Chronotherapeutic Applications of Nanotechnology

### 5.1. Nanotechnology Approaches for Glioblastoma Therapy

Nanotechnology offers promising solutions for the targeted delivery of bioactive compounds, especially in brain tumours, where the BBB is disrupted by cancer infiltration [9,10]. This technology allows therapeutic agents to reach the tumour more effectively, improving outcomes and reducing recurrence [17]. Nanoparticles may display tailored physicochemical properties, and their surface can be chemically modified to enhance biocompatibility, brain penetration, cellular uptake, and drug accumulation [9,93]. Functionalization with ligands, such as transferrin, chlorotoxin (CTX), hyaluronic acid (HA), or anti-EGFR antibodies, enables active targeting of overexpressed receptors in glioblastoma [2,11].

Among organic nanoparticles, CPP-based nanoparticles have become one of the most promising agents for intracellular delivery, mainly due to their high ability to cross cell membranes and induce uptake mechanisms [94,95]. Our group developed CPP-based protocols for the treatment of glioblastoma [12,16,75,96]. In particular, we have focused on using WRAP5 peptide-based nanoparticles functionalized with a transferrin ligand and loaded with TMZ to deliver a pDNA-coding p53 tumour suppressor to glioma cells. TMZ-WRAP5/p53 nanocomplexes mediated p53 expression and induced cancer cell apoptosis [12,96].

Polymeric nanoparticles provide controlled and sustained drug release and can be functionalized for active GB targeting [93,97]. Choi et al. developed nanoparticles based on poly (beta-amino ester) (PBAE) for delivering plasmids and for a suicide gene therapy approach aimed at treating brain cancer, promoting apoptosis and improving survival in mice [98]. Another study using a polymerized human serum albumin (HSA) nanoparticle functionalized with iRGD for the delivery of Signal Transducer and Activation of Transcription 3 factor (STAT3) siRNA effectively enhanced mice survival when combined with radiotherapy [99].

Lipid-based nanoparticles are widely studied for their biocompatibility, capacity to encapsulate both hydrophilic and hydrophobic drugs, and great ability to diffuse across cell membranes [93,97,100]. The inclusion of surface-active agents on liposomes has proven to improve their stability and ability to efficiently cross the BBB [100,101,102]. Zhao and coworkers demonstrated the ability of polymer-locking fusogenic liposome (Plofsome) to carry an interfering RNA or CRISPR-Cas9 ribonucleoprotein to glioma cells, leading to midkine (MDK) suppression and higher TMZ sensitivity without affecting healthy tissues [103]. In another study, polymetronidazole-coated verteporfin (VP) and dioleoyl-3-trimethylammonium propane (DOTAP) liposomes encapsulating a YAP small interfering inhibitor (siYAP) and functionalized with angiopep-2 successfully crossed the BBB and released siYAP and VP under hypoxia conditions, reducing tumour growth in U87 xenograft-bearing mice [104].

Inorganic nanoparticles offer physical stability and theragnostic potential [93,97,105]. TMZ-loaded magnetic nanoparticles (TMZ/MNPs-FA) combined with an external magnetic field reduced tumour mass and increased survival in rat glioma models [106]. Cobalt-doped iron oxide nanoparticles, stabilised with a carboxymethylcellulose capping ligand (Co-MION), induced apoptosis and ferroptosis in a 2D U87 brain culture and reduced tumour development and volume in 3D spheroids [107].

Biological nanoparticles provide high biocompatibility and natural targeting ability [97]. A hybrid nanoparticle composed of a Prussian Blue core and U87-derived exosome shell (Exo:PB) showed strong cellular uptake and triggered apoptosis under laser exposure in vitro, while in vivo studies demonstrated tumour accumulation and reduced volume [108]. Another hybrid system combined histidine/arginine-linked polyamidoamine (PHR) coated with exosome membranes and a T7 targeting ligand, delivering a pDNA encoding the herpes simplex virus thymidine kinase (HSVtk) [109]. Upon intravenous injection, these particles effectively targeted tumours and induced apoptosis [109].

Furthermore, recent progress in high-throughput genomic strategies and bioinformatics has improved our understanding of how genes regulate biological functions in disease, leading to the development of nucleic acid–based therapies that can target specific concerns [110,111]. Associated with nanotechnology, gene therapy has emerged as a promising approach to treat a vast range of disorders, being able to deliver exogenous nucleic acids, achieve high transfection rates, and induce gene expression, while significantly minimising toxicity, ensuring safer and more effective outcomes [94,110,112]. In the field of glioblastoma therapy, the number of published studies that aim to enhance treatment efficacy by directly addressing the genetic and molecular characteristics of GB has been increasing over time. Yuan et al. developed peptide/siEGFR/siP65 for the dual silencing of *EGFR* and *RELA/P65* in a GB mouse model, leading to remarkably increased survival without noticeable toxicity when combined with radiotherapy [113]. Another lipid nanocarrier modified with angiopep-2 peptide was able to effectively cross the BBB in a GB mouse model, accumulate within the brain tumour and silence the polo-like kinase 1 (*PLK1*), resulting in tumour growth inhibition and increased mice median survival [114]. In a phase I/II clinical trial, WT1-mRNA/dendritic cell vaccination was well accepted, with no detectable systemic toxicity [115]. Vaccination led to disease control and a higher median OS [115]. Collectively, these already-published studies support the potential of nanotechnology combined with gene therapy to improve treatment efficacy and reduce off-site effects.

**Figure 5:**
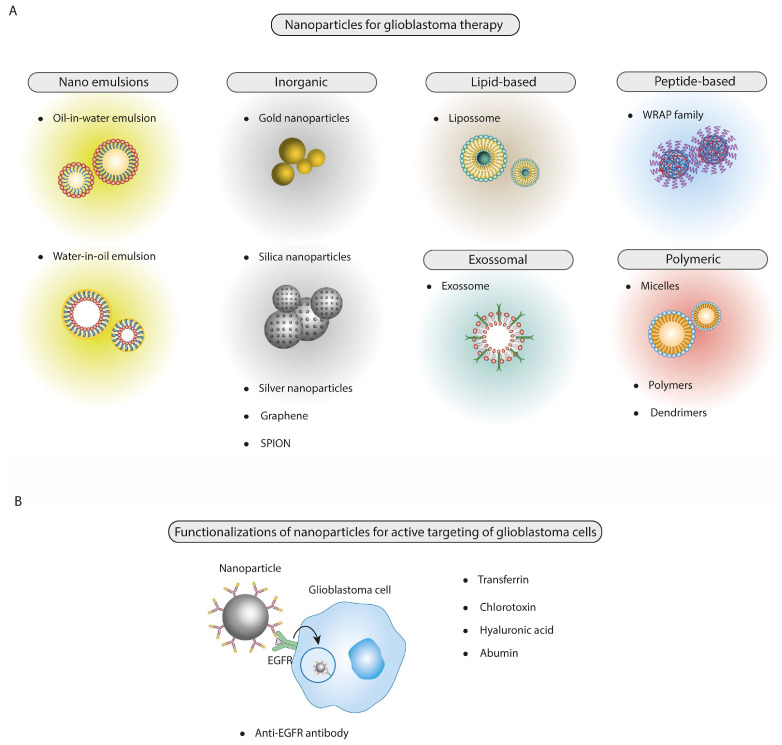
Nanotechnology-based approaches to improve glioblastoma therapy: (**A**) Overview of major nanoparticle platforms explored for glioblastoma therapy, including nanoemulsions, inorganic particles, liposomes, and exosomes. (**B**) Nanoparticle functionalization strategies for active targeting of glioblastoma cells, highlighting the use of certain targeting molecules for highly expressed receptors in the BBB and glioblastoma cells. This figure was partially composed using images retrieved from Servier Medical Art (Servier), licenced under the Creative Commons Attribution 4.0 International License (CC BY 4.0), Available online: https://creativecommons.org/licenses/by/4.0/ (accessed on 3 February 2026). SPION—Superparamagnetic iron oxide nanoparticles; EGFR—Epidermal growth factor receptor.

Nanotechnology offers promising strategies to enhance the efficacy and precision of glioblastoma therapy, minimising side effects, and overcoming treatment resistance. The following figure, Figure 5, provides an overview of the main nanoparticle platforms used in glioblastoma treatment, as well as the strategies employed to actively target the BBB and tumour cells through surface modifications.

### 5.2. Integrating Nanotechnology with Chronobiology for Optimised GB Therapy

Nanotechnology has transformed drug delivery by enabling the targeted, controlled, and precise delivery of drugs. Conventional drug delivery approaches typically fail to account for the body’s natural rhythms, maintaining constant drug levels and potentially reducing therapeutic effectiveness [56]. Crucially for chronotherapy, the temporal precision of drug release used to modulate the molecular clock or optimise drug administration, as provided by nanotechnology, aligns well with the principles of circadian medicine, creating more effective, tolerable, and personalised treatments [56]. The goal is to maximise therapeutic efficacy by administering drugs when target pathways are more responsive, when disease activity reaches its peak, or when the patient’s physiological tolerance to treatment is greatest [56]. Importantly, emerging evidence suggests that the uptake and transport mechanisms of delivery systems across the BBB seem to be under the influence of circadian rhythms, further emphasising the relevance of temporal optimisation. Among the different strategies, systems capable of temporally controlled release and receptor-targeted delivery across the BBB appear particularly promising, as they directly align with circadian modulation of both disease activity and barrier permeability. Several receptors and transporters present in the BBB have shown rhythmical expression and functional circadian patterns, and/or their expression has been found to be influenced by clock genes [70,97,116]. Therefore, functionalizing nanoparticles with ligands directed at receptors under circadian regulation may provide a strategy to increase neural tissue-specific delivery [117]. For instance, N-methyl-D-aspartate receptor (NMDAR) has been shown to regulate central clock and clock genes expression and the low affinity neurotrophin receptor p75^NTR^ present in brain astrocytes involved in glucose and glycogen metabolism was proved to be subject to regulation by the BMAL1/CLOCK complex [118,119]. 

Despite the evidence, the intersection between nanotechnology and chronobiology has received little attention to date [56,67,68,69,70,97]. The intricate nature of human circadian rhythms and the lack of reliable circadian biomarkers, along with individual chronotypes and variations on physiological conditions, are among the obstacles that make it difficult to maintain consistent therapeutic outcomes and clinical adoption [31]. Additionally, many preclinical studies rely on in vitro models that cannot fully replicate the complex tumour or CNS microenvironment, and significant differences exist between rodent and human circadian rhythms, which may impact the translational relevance of timing-dependent effects [31]. Protocols for circadian synchronisation also vary widely between studies, potentially contributing to variability in results. Nevertheless, not taking into account rhythmic variations may result in suboptimal drug exposure at specific times, potentially diminishing the efficacy of treatments [56]. Approaches that integrate both circadian timing and active targeting mechanisms may offer advantages over delivery systems that neglect temporal variability, while acknowledging that further work in clinically relevant models is needed to confirm and optimise these strategies. As far as the authors are aware, only one experiment studied the influence of circadian clockwork on nanocarrier uptake, aiming to optimise nanoparticle-based therapy. Goraya’s group studied the influence of daily patterns on PLGA-b-HA nanoparticle uptake, adhesion, and retention [120]. The employment of a computational model, in conjunction with fluctuations in circadian blood flow in an in vivo arterial model, has substantiated the notion that circadian variations in flow velocity and blood pressure exert a significant influence on nanoparticle retention, with a concomitant increase observed during the sleep cycle [120]. Nevertheless, some studies have linked the performance of drug-carrying nanoparticles to chronotherapy to treat other types of cancer and highlighted the importance and feasibility of chrono-modulated delivery [121,122]. A study investigating the impact of the circadian clock on G4-PTX-R8 conjugated-PAMAM dendrimer cellular uptake and therapeutic efficacy in human cervical cancer cells (HeLa) found a pronounced cell uptake in the nanosystems at time points T8 and T12 (indicating 8 and 12 h post-synchronisation), which led to higher apoptosis levels [123]. These results suggest a time-specific regulatory mechanism influencing the performance of these systems. Moreover, the knockout of *Bmal1* affected paclitaxel and caspases rhythmic expression patterns, and caspase-3 expression [123].

In addition to glioblastoma, nanomedicine has been investigated in the context of circadian rhythms to enhance therapeutic precision in other CNS pathologies, such as sleep disorders and cancer [117]. In an in vivo study published in 2025, Gao et al. developed a composite nanoparticle system for the targeted delivery of 5-hydroxytryptophan (5-HTP) to the brain in a mouse model of sleep-deprivation-induced insomnia [124]. Following administration, treated mice exhibited improved cognitive performance and normalisation of circadian rhythm-related gene expression (including *Per*1, *Per*2, and *Cry*1) [124]. These findings indicate that sleep deprivation disrupts circadian regulation, which can be partially restored and modulated through nanoparticle-mediated 5-HTP delivery. In a cancer model, a nanoplatform incorporating the IR-820 photothermal sensitiser and prodrug components was used to manipulate tumour circadian oscillators in ovarian cancer [125]. By controlling the efficacy of chemotherapeutic agents with near-infrared light, which synchronises circadian disruption with drug action, the authors achieved robust tumour apoptosis [125].

**Figure 6:**
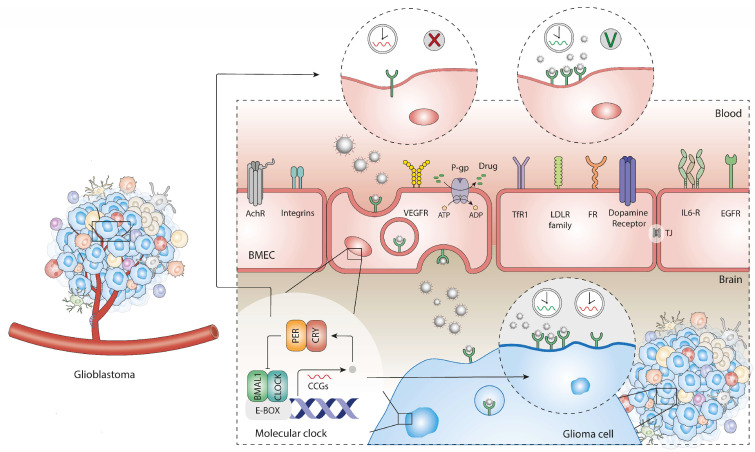
Chrono-nanotherapy strategies for targeted glioblastoma treatment. This figure illustrates how circadian regulation influences BBB dynamics, including variations in surface receptor expression and tight junction integrity. These fluctuations influence nanoparticle uptake and, consequently, the therapeutic efficacy of treatment, highlighting the potential of timing-based approaches to optimise glioblastoma nanotherapy. This figure was partially composed using images retrieved from Servier Medical Art (Servier), licenced under the Creative Commons Attribution 4.0 International License (CC BY 4.0), Available online: https://creativecommons.org/licenses/by/4.0/ (accessed on 4 February 2026). BMEC—Brain microvascular endothelial cell; AchR—Acetylcholine receptor; VEGFR—Vascular endothelial growth factor receptor; ATP—Adenosine triphosphate; ADP—Adenosine diphosphate; P-gp—P-glycoprotein; TfR—Transferrin receptor; LDLR—Low-density lipoprotein receptor; FR—Folate receptor; IL6-R—Interleukin 6 receptor; EGFR—Epidermal growth factor receptor; CCG—Clock-controlled genes.

Therefore, the integration of chronobiology with nanotechnology by developing appropriate nanoparticles for the precise and controlled release of anticancer drugs whose performance is synchronised with individual circadian rhythms may be a strategy to improve cancer treatment outcomes, particularly for diseases such as glioblastoma that exhibit pronounced circadian dysregulation [56]. Figure 6 illustrates how nanotherapy can be synchronised with the body’s biological rhythms to improve glioblastoma treatment. It demonstrates the influence of timing on blood–brain barrier permeability, receptor expression, nanoparticle delivery, and therapeutic outcomes.

Nanotechnology provides powerful tools for glioblastoma therapy by enabling targeted, controlled, and efficient delivery of drugs and genetic material across physiological barriers. Functionalized nanoparticles are capable of enhancing cellular uptake, tumour specificity, and therapeutic efficacy while minimising off-target effects. Importantly, integrating nanotechnology with circadian principles allows for time-tuned drug release. Although still in early stages, chrono-nanomedicine represents a promising strategy for personalised, precise, and more effective glioblastoma treatments, with potential applicability to other CNS pathologies.

Despite the promise of chronotherapy and nanotherapy integration into GB treatment, several practical challenges must be addressed for successful clinical implementation. Accurate assessment of individual patient chronotypes is essential to tailor treatment timing, but can be logistically complex. Patient adherence to precisely timed drug administration and radiation schedules may be difficult to maintain outside controlled clinical settings. Hospital workflow and staffing must be coordinated to allow time-specific interventions, and regulatory hurdles remain for the approval of novel nanoformulations, multi-agent combinations, and devices designed for temporally controlled delivery. Addressing these challenges will require interdisciplinary collaboration, patient-centred strategies, and integration of technological solutions such as responsive/smart drug delivery systems and AI-guided treatment scheduling to ensure safe, feasible, and effective chrono-nanotherapy.

#### Circadian-Responsive Delivery Systems

Smart delivery systems represent an important technological advance in this field, enabling precise temporal control for effective chronotherapy. In response to specific internal/physiological stimuli (pH, presence of specific molecules, molecule concentration, redox state, etc.) or external cues (light, magnetic, temperature, ultrasound), these delivery systems release cargo when required [56]. Furthermore, these systems can be designed to respond to endogenous circadian-regulated variations in receptor expression, barrier function, or cellular metabolism, triggering cargo release when required. Functionalized delivery systems for receptors such as NMDAR, p75^NTR^, GLUT1, or P-gp, which have been shown to possess time-dependent fluctuations and circadian patterns, can enhance uptake and therapeutic efficacy at specific times [56].

As an example of a chrono-optimised delivery system, a study using upconverted nanoparticles coated with poly(acrylic acid) (PAA) and a UV-cleavable nitrobenzyl photolinker achieved complete TMZ release into U87 glioblastoma cells within 30 min, inducing cell death and demonstrating precise spatiotemporal control over drug release [126]. Other studies have highlighted the versatility and efficacy of smart nanoparticle-based delivery systems in cancer treatment. Yuan et al. developed hollow mesoporous Prussian Blue@zinc phosphate nanoparticles (HMPB-GOx@ZnP-DOX) for co-delivery of doxorubicin and glucose oxidase that respond to endogenous tumour pH and photothermal stimuli, achieving strong tumour inhibition in vivo without systemic toxicity [127]. Xiao et al. developed hydrogen peroxidase- and pH-responsive boronate ester lipid nanoparticles that co-deliver a platinum prodrug and veliparib, achieving enhanced breast cancer cell apoptosis in vitro [128]. These studies highlight the potential of stimulus-responsive smart delivery systems to deliver chemotherapeutics with exceptional precision and, thus, enhanced therapeutic efficacy.

## 6. Conclusions

Despite intensive research efforts aimed at improving GB treatment outcomes, only modest advances have been made. GB is still a highly aggressive and deadly form of brain cancer with a poor prognosis. This fact emphasises the urgent need for more effective and personalised treatment options. As shown in this review, circadian clock components may play an important role in the mechanisms underlying GB pathogenesis and can serve as potential molecular targets for the development of new clinical protocols. The circadian clock has the ability to modulate oncogenic processes, and cancer cells can, in turn, reprogram the clock to promote their viability [32]. The importance of integrating circadian clock biology with the administration of certain drugs (TMZ, 1A-116, Bortezomib), adjuvants (RORα, CRY agonists) or nanoparticles (or ideally a combination of these strategies) seems now to be crucial to delay GB progression, improve drug outcomes, increase survival, and reduce the associated effects [1,3]. In fact, clock proteins seem to have an impact on cells’ sensitivity to chemotherapies and radiation, and their modulation can lead to a reduction in the resistance that is often associated with current treatments. The implications of circadian clock regulation on BBB permeability also open promising possibilities for the future of chronotherapy in GB. Given the limited number of studies, future research is needed to understand the complex interactions among circadian clock regulation, multi-agent therapeutic schedules, BBB permeability, and GB pathophysiology. To optimise these efforts, experimental designs should incorporate inter-individual variability, multiple chronotypes, and clinically relevant in vivo models. Identification and validation of circadian biomarkers, including clock gene expression profiles, melatonin rhythms, and actigraphy-based measurements, will be essential to stratify patients and personalise treatment schedules [56]. Clinical trials should adopt frameworks that allow the precise timing of drug/gene administration, the integration of multi-agent therapies, and the evaluation of both pharmacokinetic and pharmacodynamic outcomes within the context of circadian rhythms.

Chronotherapy approaches strengthened by progress in computational tools (artificial intelligence, machine learning) and gene-editing technologies are gaining scientific attention, as these tools can monitor, predict, and adjust treatment timing according to individual circadian profiles and the time at which a disease will benefit from a particular treatment. This approach supports personalised chronotherapy, as treatment administration can be tailored to the patient’s unique circadian rhythm-related gene expression signatures, potentially improving efficacy and reducing side effects. In parallel, the development of smart drug delivery systems designed to improve brain targeting and temporal control release, further aligned with patient-specific rhythms, is also gaining interest. These platforms are non-invasive, can be designed to be highly responsive, and are capable of modulating drug release profiles in ways that can be aligned with circadian rhythms. In the future, delivery systems will not only improve transport into the brain but will also release therapeutics in a temporally controlled manner, synchronising with individual circadian patterns.

With the increasing identification of core clock molecules’ involvement in GB pathogenesis, future research should not only integrate chronotherapy into the development of new therapeutic protocols but also explore the potential of combining different strategies, such as targeted therapy, immunotherapy, and nanotechnology-based approaches, administered in a circadian-timed manner. Precise validation and effective implementation of such circadian-based protocols in translational models will be essential for successful integration into the clinic. Large-scale clinical trials, healthcare professional training, and broad patient access to technologies will be crucial for validating the safety and effectiveness of personalised chronotherapy and for integrating circadian medicine into healthcare systems.

## 7. Future Directions

Although the strategy described above holds considerable potential, translating it from the laboratory to the clinic faces significant challenges. Many preclinical studies rely on in vitro or rodent models, which may not fully replicate the human tumour microenvironment, barriers, intrinsic circadian fluctuations, the intricate nature and complexity of human circadian rhythms, or inter-individual variability in circadian rhythms [56,116,129]. Circadian phenotypes, timing schedules, and differences in synchronisation protocols can also affect outcomes, potentially limiting the translational relevance of these approaches. Therefore, while experimental data are encouraging, cautious interpretation is required, and further studies in clinically relevant models are essential before clinical implementation. Overcoming these obstacles is paramount for successful integration into standard clinical practice. One promising way to achieve this is by developing nanotechnology-based protocols that use approved drugs that are known to modulate circadian clock components (and whose safety profiles and pharmacokinetics are already well established) and integrating them into chronotherapy [56]. More recently, high-throughput screening combined with advanced computational tools has enabled the characterisation of circadian profiles and the design of new small molecules capable of modulating specific components of the circadian clock [56,130]. In parallel, artificial intelligence models (including deep learning, machine learning, and hybrid mechanistic-data approaches) have been applied to analyse real-time rhythmic phase shifts, circadian biomarkers, amplitude reductions, and desynchronizations using large datasets from wearable sensors, genetic research, actigraphy, clinical trials, and biomarker measurements [129,130,131]. These models hold significant potential for chronotherapy by predicting circadian patterns and the optimal timing for GB treatment schedules [130]. Interdisciplinary collaboration among chronobiologists, computational scientists, and clinicians in designing these models should be a priority [131]. Several studies have demonstrated promising results in the use of predictive models based on circadian rhythm gene profiles that are used to predict GB prognosis, potential biomarkers, and therapeutic targets, promoting accurate treatment [132,133,134,135,136]. Building on this, future research should focus on the use of artificial intelligence models to identify patient-specific circadian profiles and to design and monitor smart drug delivery systems that release therapeutics in synchrony with these individual rhythms. Gene editing tools (CRISPR/Cas9) and gene therapy offer powerful platforms to restore and enhance the circadian clock [130,137,138]. By selectively knocking out, activating, or modifying core clock genes, such as *BMAL1*, *CLOCK*, *PER*, or *CRY*, researchers can investigate how circadian disruption shapes GB tumour physiology, pathology, and therapeutic sensitivity. In the context of nanotechnology and brain-targeted delivery, gene regulation can enhance responsiveness and impact therapeutic success. Nevertheless, ethical and regulatory considerations (such as patient compliance, data privacy, and the risks of targeting the core clock components), scalability, equity, and cost-effectiveness remain questions surrounding the integration of nanotechnology, chronotherapy, and artificial intelligence into clinical practice [56,131]. Thus, to optimise the clinical application of chronotherapy and nanomedicine, future studies should focus on:

- How circadian mechanisms affect BBB permeability, and therefore, the delivery of anti-cancer agents to cancer cells [139];
- Elucidating the crosstalk between the central and peripheral circadian clocks and other key physiological mechanisms, including the immune and metabolic pathways [56,130];
- Considering animal models with different chronotypes;
- How chronotherapeutic interventions may affect the overall cellular circadian rhythm and other clock pathways [3];
- Readjusting the circadian clock with external zeitgeber patterns, such as light and food, as a study on *Drosophila* showed that the re-adjustment of their diurnal cycle delayed GB progression and reduced related adverse consequences [140];
- Investigating ultradian and infradian rhythms, which have been shown to influence treatment response in GB patients [3];
- Including a comprehensive profile of each patient’s circadian clock-related gene expression, environmental cues, and lifestyle patterns, to support personalised diagnosis and treatment [28];
- The timing of administration or irradiation, as pharmacokinetics and pharmacodynamics, should be studied according to patients’ chronotypes to reduce the number of doses needed and the consequent adverse effects [28];
- The timing of sample collection and the daily habits of patients;
- The age, genetics, and sex of patients as factors that may confound the results [3];
- The alignment of a patient’s internal circadian rhythm with the external environment. This includes maintaining robust light/dark cycles and minimising nocturnal exposure to blue light—especially from artificial sources such as LED lights, screens, and electronic devices—due to their impact on melanopsin-expressing retinal ganglion cells and subsequent SCN entrainment.

Integrating circadian biology into GB treatment will require an interdisciplinary approach that combines a deep understanding of molecular rhythms, predictive modelling, and personalised treatment strategies while considering each patient’s unique characteristics and environmental cues. By addressing the outlined challenges and systematically incorporating circadian principles into safe and effective clinical practice, we have the opportunity to reshape GB therapy.

## Figures and Tables

**Table 1:** List of chronotherapy strategies in glioblastoma.

Therapy	Model Type	Major Findings	Reference
TMZ chemotherapy	In vitro	- MGMT promoter methylation presented daily rhythms, and high MGMT levels matched with maximal TMZ efficacy - A theoretical model suggested higher TMZ-induced apoptosis in early morning	[83]
	In vitro/In vivo: GB mouse orthotopic model	- GB cells, MGMT, and promoter methylation presented intrinsic circadian rhythms - Higher TMZ susceptibility coincided with BMAL1 peak - MGMT inhibition abrogated daily rhythm sensitivity to TMZ - Morning TMZ administration decreased tumour size in mice	[84]
	Clinical trial	- TMZ intake according to each patient’s biological clock can impact chemotherapy efficacy	[85]
Metformin combined with radiotherapy	In vitro	- Metformin promoted PER2 expression and SIRT2/G6PD signalling pathway inhibition, enhancing radiotherapy sensitivity and cell growth inhibition	[86]
Dexamethasone (DEX)	In vivo: GB mouse orthotopic model	- Glucocorticoid signalling was observed to possess daily rhythms and to be modulated by *BMAL1* and *CRY* - DEX time administration influenced GB progression	[87]
Anti-PD-1/TMZ		- Anti-PD-1 monotherapy alone or combined with TMZ originated an immune oscillatory response pattern	[88]
Melatonin		- Melatonin significantly inhibited tumour growth and reduced vascularization - Under constant light exposure, circadian rhythms were supressed, and melatonin partially restored rhythmicity	[89]
*Lycium barbarum* (LbGP) extract		- LbGP significantly inhibited the proliferation of GB cells and supressed lipogenesis via PER2 - LbGP up-regulated PER2, which silencing abolished LbGP effects	[90]

## Data Availability

No new data were created or analyzed in this study.

## Abbreviations

The following abbreviations are used in this manuscript:

5-HTP	5-Hydroxytryptophan
AchR	Acetylcholine receptor
ADP	Adenosine diphosphate
Akt	Protein kinase B
ARF	ADP-ribosylation factor
ATP	Adenosine triphosphate
B cell	B lymphocyte
BBB	Blood–brain barrier
BCNU	Carmustine
BMAL1	Basic helix-loop-helix ARNT-like protein 1
BMEC	Brain microvascular endothelial cell
CAR-T	Chimeric antigen receptor T-cell
CCG	Clock-controlled genes
CCNU	Lomustine
CDK	Cyclin-dependent kinase
CLOCK	Circadian locomotor output cycles protein kaput
CM-NP	Curcumin-loaded noisome nanoparticle
CNS	Central nervous system
Co-MION	Carboxymethylcellulose capping ligand
CPPs	Cell-penetrating peptides
CRY	Cryptochrome
CTX	Chlorotoxin
DBP	D-box binding protein
DEX	Dexamethasone
DOTAP	Dioleoyl-3-trimethylammonium propane
Dp44mT	Di-2-pyridylketone 4,4-dimethyl-3-thiosemicarbazone
ECM	Extracellular matrix
ECs	Endothelial cells
EGFR	Epidermal growth factor receptor
FDA	Food and Drug Administration
FR	Folate receptor
GB	Glioblastoma
GDP	Guanosine diphosphate
GEF	Guanine nucleotide exchange factor
GSCs	Glioblastoma stem-like cells
GSK-3	Glycogen synthase kinase-3
GTP	Guanosine triphosphate
HA	Hyaluronic acid
HeLa	Human cervical cancer cells
HIF-1α	Hypoxia-inducible factor 1-alpha
HSA	Human serum albumin
HSVtk	Herpes simplex virus thymidine kinase
ICI	Immune checkpoint inhibitors
IDH-1	Isocitrate dehydrogenase 1
IL-1β	Interleukin-1ß
IL6-R	Interleukin 6 receptor
INK	Cyclin-dependent kinase inhibitors
LbGP	*Lycium barbarum*
LDHA	Lactate dehydrogenase A
LDLR	Low-density lipoprotein receptor
LGMN	Legumain
LIC	Lactate-IL-1β-Clock
MDK	Midkine
MDM2	Mouse double minute 2 homologue
MDMX	Mouse double minute X homologue
MDSCs	Myeloid-derived suppressor cells
MGMT	O6-methylguanine-DNA methyltransferase
MRI	Magnetic resonance imaging
NDRG	N-myc downstream-regulated gene
NFIL	Nuclear factor, interleukin 3-regulated.
NF-Κb	Nuclear factor-κB
NK	Natural killer
NMDAR	N-methyl-D-aspartate receptor
NSCs	Neural stem cells
OS	Overall survival
PAA	Poly(acrylic acid)
PBAE	Poly(beta-amino ester)
PCV	Vincristine
PDGFR	Platelet-derived growth factor receptor
PER	Period
PFS	Progression-free survival
P-gp	P-glycoprotein
PHR	Histidine/arginine-linked polyamidoamine
PI3K	Phosphoinositide 3-kinase
PIP	Phosphatidylinositol phosphate
PLK1	Polo-like Kinase 1
Plofsome	Polymer-locking fusogenic liposome
pRB	Retinoblastoma protein
PTEN	Phosphatase and tensin homologue
RAR	Retinoic Acid Receptor
RAS	Rat sarcoma small GTPase
REV-ERB	Nuclear Receptor Subfamily 1 group D
ROR	RAR-related Orphan Receptor
RORE	Retinoic acid-related orphan receptor response element
RTK	Receptor tyrosine kinase
SCN	Suprachiasmatic nucleus
Sev	Sevoflurane
siYAP	YAP small interfering inhibitor
SPION	Superparamagnetic iron oxide nanoparticles
STAT3	Signal transducer and activator of transcription 3
T cell	T lymphocyte
TfR	Transferrin receptor
TIAM1	Metastasis-inducing protein-1
TME	Tumour microenvironment
TMZ	Temozolomide
TTFL	Translational-transcriptional feedback/feed-forward loops
VEGF	Vascular endothelial growth factor
VEGFR	Vascular endothelial growth factor receptor
VP	Verteporfin
WHO	World Health Organization
